# Fluid bolus therapy in pediatric sepsis: a narrative review

**DOI:** 10.1186/s40001-022-00885-8

**Published:** 2022-11-12

**Authors:** Julian San Geroteo, Michaël Levy, Julien Gotchac, Olivier Brissaud, Stéphane Dauger

**Affiliations:** 1 Pediatric Intensive Care Unit, Robert-Debré University Hospital, Paris, France; 2 Université Paris Cité, Paris, France; 3 Neonatal and Pediatric Intensive Care Unit, Children’s University Hospital, Bordeaux, France

**Keywords:** Pediatric sepsis, Children, Fluid bolus therapy, Balanced crystalloids, Surviving sepsis campaign

## Abstract

Leading cause of death in children under five, pediatric sepsis remains a significant global health threat. The 2020 Surviving Sepsis Campaign guidelines revised the management of septic shock and sepsis-associated organ dysfunction in children. In addition to empiric broad-spectrum antibiotics, fluid bolus therapy is one of the cornerstones of management, due to theoretical improvement of cardiac output, oxygen delivery and organ perfusion. Despite a very low level of evidence, the possible benefit of balanced crystalloids in sepsis resuscitation has led to discussion on their position as the ideal fluid. However, the latest adult data are not consistent with this, and the debate is still ongoing in pediatrics. We provide here the current state of knowledge on fluid bolus therapy in pediatric sepsis with emphasis on balanced crystalloids.

## Introduction

Pediatric sepsis incidence and mortality are, respectively, 25.2 million and 3.4 million worldwide in 2017. More than half of sepsis cases and a third of sepsis-related deaths worldwide occur in children particularly in those under 5 years of age [1]. A recent large European prospective study of 2844 children with life-threatening infections reported a median age of 28 months in the cohort of 1229 pediatric sepsis [2]. The global case-fatality rate of pediatric sepsis is about 13% subjected to geographic variations [3, 4]. Sepsis accounts approximately for 3–8% of Pediatric Intensive Care Unit (PICU) admissions [5, 6]. Microbiological confirmation was obtained in only half of children, bacterial in about 45% of cases [2]. Two thirds of sepsis occurs in children with chronic disease who have higher in-hospital mortality [7].

To date, pediatric sepsis definition reference remains that of the 2005 consensus conference, which classified sepsis as infection in presence of systemic inflammatory response syndrome [8]. The lack of sensitivity and specificity of these historical criteria [9, 10] led to a change in the adult definition of sepsis as a “life-threatening organ dysfunction caused by a dysregulated host response to infection” [11]. However, this concept is not yet validated in pediatrics [12], and therefore, the latest guidelines of the Surviving Sepsis Campaign refer to children with infection and cardiovascular (i.e. “septic shock”) or non-cardiovascular organ dysfunction (i.e. “other sepsis-associated organ dysfunction”) [13].

Sepsis bundles include an early fluid resuscitation, the obtention of a quick vascular access, collection of blood cultures, rapid initiation of broad-spectrum antibiotics, the measure of lactate and early administration of vasoactive agents if shock persists [13]. Completion of sepsis bundles lead in decreasing sepsis-related mortality [14].

Although the use of normal saline remains predominant in pediatric sepsis [15, 16], balanced crystalloids are now suggested as the first-line fluid in the latest guidelines of the Surviving Sepsis Campaign [13] and the European Resuscitation Council [17]. However, the ideal fluid debate is still ongoing [18]. In this narrative review, we aim to provide an overview of the current state of knowledge regarding fluid bolus therapy in pediatric sepsis, with emphasis on available data regarding balanced crystalloids.

## Fluid bolus therapy indications

World Health Organization (WHO) recommendation in the 1990s to perform aggressive fluid bolus therapy reduced tenfold the mortality of pediatric sepsis [19]. Fluid bolus therapy improves cardiac output, oxygen delivery, and organ perfusion in sepsis [20], although such a response is variable and unpredictable [21, 22]. Fluid bolus therapy indications in pediatric sepsis have recently been revised in the Surviving Sepsis Campaign [13].

In healthcare systems with access to intensive care, fluid bolus therapy is recommended in case of abnormal perfusion or hypotension for age. Clinical signs of poor tissue perfusion are tachycardia, altered mental status, decreased urine output (below 1 ml/kg/h) and abnormalities in capillary refill time (flash < 1 s or prolonged > 3 s), skin (mottled, cool or flushed, ruddy) and pulses (weak or bounding) [23]. Despite being a standard of care in high income countries, the evidence for fluid bolus therapy in pediatric sepsis is limited in these settings [24] and is based on historical studies of weak methodology [25].

In healthcare systems without access to intensive care, fluid bolus therapy is only recommended in case of hypotension, defined as low systolic blood pressure (i.e., < 50 mmHg before 1 year, < 60 mm Hg between 1 and 5 years and < 70 mm Hg after 5 years) or all three WHO shock criteria (i.e., cold extremities, capillary refill time greater than 3 s and weak or fast pulse) [13]. This major distinction regarding fluid bolus therapy indications in low- and middle-income countries, where intensive care is not routinely accessible, comes mainly from the FEAST (*Fluid Expansion As Supportive Therapy*) study, the only randomized clinical trial (RCT) to date that has evaluated fluid bolus therapy in pediatric sepsis. Maitland et al. randomized 3141 sub-Saharan African children aged 2 months to 12 years with sepsis and abnormal perfusion without severe hypotension to, either fluid bolus therapy (20 mL/kg NaCl 0.9% or 5% albumin) or no bolus with maintenance fluid only. After exclusion of severe malnutrition or gastroenteritis, mortality at 48 h and 1 month was higher with fluid bolus therapy. However, the findings of the FEAST should be interpreted cautiously [26, 27]. Indeed, the WHO shock criteria were absent in one third of children and lack of PICU availability worsens the outcome of fluid-related complications. Moreover, a transfusion threshold of 5 g/dL, not reached in one third of cases, and the diagnosis of malaria in more than half of the cases limit the external validity of this study in high resource settings, especially since fluid bolus therapy could be dangerous in these situations because of hemodilution [28].

Finally, in both healthcare systems, fluid bolus therapy should be discontinued as soon as signs of fluid overload develop, such as pulmonary oedema or new or worsening hepatomegaly. Moreover, cumulative positive fluid balance is associated with poor outcomes in children [29, 30], especially with sepsis [31–33].

In summary, according to the Surviving Sepsis Campaign, fluid bolus therapy indications in pediatric sepsis are limited to hypotension, regardless of health systems, and abnormal perfusion (only in settings with availability of intensive care) [13]. Once the decision for volume expansion has been made, one of the key elements that may affect children’s outcome, is the fluid choice for sepsis initial resuscitation.

## Resuscitation fluid choice: crystalloid solutions

## Rationale

Composed of water and electrolytes, crystalloid solutions (Table 1) are divided in normal saline (or NaCl 0,9% and “physiological” saline) and balanced crystalloids. Normal saline is a high-chloride solution with 154 mmol/L of both Na + and Cl-, main cation and anion of the body, respectively. Balanced crystalloids are low-chloride solutions, closer to the electrolyte composition of plasma, where Cl- anions are replaced by buffers (lactate, acetate, gluconate, or malate), quickly excreted, or metabolized into bicarbonate. According to Stewart approach, acid–base balance is mainly determined by the strong ion difference, i.e. the difference between all fully dissociated cations ([Na^+^] + [K^+^] + [Ca^2+^] + [Mg^2+^]) and anions ([Cl^−^] + [lactate] + [SO_4_^2^]) [34]. Physiologically, plasma strong ion difference is about 40 mmol/L (total strong cation > strong anion concentrations). Below 24 mmol/L, the lower the strong ion difference, the more dissociation of H_2_0 into H^+^ and OH^−^ occurs, leading to hyperchloremic metabolic acidosis. Because of its zero SID ([Na^+^] = [Cl^−^]), the use of normal saline drives to a lower bicarbonate concentration, a shift of H^+^ and K^+^ out of cells for electroneutrality and hyperchloremic metabolic acidosis. Balanced crystalloids have strong ion difference > 24 mmol/L with a neutral or alkalizing action on pH, due to lower Cl- concentration than Na^+^ [35, 36].

**Table 1:** Composition of commonly used crystalloids

	Human plasma	Normal saline	Hartmann’s solution	Ringer’s lactate	Ringer’s acetate	Plasma-Lyte	Sterofundin
*Sodium*	140 ± 5	154	131	130	130	140–141	145
*Chloride*	102 ± 8	154	111	109	112	98	127
*Potassium*	4.5 ± 1	0	5	4	5	5	4
*Calcium*	2.4 ± 0.2	0	2	1.4	1	0	2.5
*Magnesium*	0.9 ± 0.1	0	0	0	1	1.5	1
*Bicarbonate*	28 ± 4	0	0	0	0	0	0
*Lactate*	< 2	0	29	28	0	0	0
*Gluconate*	0	0	0	0	0	23	0
*Acetate*	0	0	0	0	27	27	24
*Malate*	0	0	0	0	0	0	5
*Osmolarity*	285 ± 10	308	278	273	276	295	309
*pH*	7.4 ± 0.02	4.5–7.0	5.0–7.0	6.0–7.5	6.0–8.0	6.5–8.0	5.1–5.9
*SID*	40 ± 2	0	27	27	25	50	27
*Na/Cl ratio*	1.21–1.54	1	1.18	1.19	1.16	1.43	1.14

All variables are expressed in mmol/L except for osmolarity (mosm/L) and pH. *SID* strong ion difference

Hyperchloremia decreases tubular chloride reabsorption, resulting in arteriolar vasoconstriction, and therefore, a decrease in glomerular filtration rate and urine output [37]. Apart from the kidney, chlorine-rich fluids are associated with increased inflammation, coagulopathy, macrocirculatory and microcirculatory haemodynamic alterations [38].

Hyperchloremia and hyperchloremic metabolic acidosis are common in PICU, occurring in 95% and 39% of children, respectively [39]. Hyperchloremia or even moderate increase in [Cl^−^] ≥ 5 mmol/L has been associated with poor outcomes in children in several retrospective studies [40–43].

The Surviving Sepsis Campaign recommends, with very low quality of evidence, balanced crystalloids rather than normal saline, as the first-line for fluid bolus therapy in pediatric sepsis [13]. The latest guidelines of the European Resuscitation Council have recently extended this recommendation to all childhood circulatory failures [17].

In children without sepsis, a recent meta-analysis of Lehr et al. (13 studies, 11,848 children) comparing balanced crystalloids (Ringer’s Lactate in 8/13 studies) and normal saline showed a significant improvement regarding pH and serum bicarbonate level (mean increase of 0.03 and 1.6 mmol/L, respectively) with balanced crystalloids in the pooled analysis of 3 RCTs with acute gastroenteritis. No differences were found in metabolic acidosis, hyperchloremia, acute kidney injury, need for mechanical ventilation, vasopressor, or renal replacement therapy, as well as PICU or hospital length of stay and mortality. Interpretation of this work is difficult because of the multiple analyses performed on studies with small sample sizes, highly heterogeneous patients and end-points, two of which were at high risk of bias [44].

In pediatric sepsis, a recent Indian RCT of 708 children with septic shock found a reduction in the incidence of acute kidney injury, need for renal replacement therapy and hyperchloremia but not modification on survival with Plasma-Lyte [45]. Most of the other existing data are from two large American retrospective studies with equivocal results. Emrath et al. found a reduction in mortality, acute kidney injury and need for vasopressors with balanced crystalloids at 72 h in 7000 children [46], whereas Weiss et al. showed no difference on acute kidney injury or mortality between normal saline and balanced crystalloids in 4234 pediatric sepsis but increase hospital length of stay with balanced crystalloids. Nevertheless, in this last study, the severity of children who received balanced crystalloids (older and statistically greater age, crystalloid volume, cardiovascular and respiratory disease, steroids, albumin, and transfusion), and the small number of children (459/4234) who received “only balanced crystalloids” suggests that this was a second-line treatment and makes the comparison difficult. In addition, the median volume of crystalloid of 24 ml/kg, may have diminished the power of the study [47]. Finally, a recent observational study of 99 pediatric sepsis found a decrease in acute kidney injury, need for renal replacement therapy and hospital length of stay with balanced crystalloids [48]. Thus, the debate is not over and the results of an ongoing international RCT with anticipated enrollment of 8,800 children with septic shock are eagerly awaited [49], especially as two recent large RCTs in adults ICUs did not report improved survival with balanced crystalloids [50, 51].

### Clinical practice

In clinical practice, as Ringer’s Lactate is slightly hypotonic (Table 1), others balanced crystalloids or normal saline should be used instead in patients at risk of increased intracranial pressure (e.g., traumatic brain injury, diabetic ketoacidosis). Normal saline should be preferred in hypovolemic hyponatremia or hypochloremic contraction with metabolic alkalosis due to its higher [Na^+^] and [Cl^−^] content. In all other situations, balanced crystalloids are suggested as first-line crystalloids, especially in pediatric sepsis [13, 17]. They should be used with caution in cases of anuria, although hyperkaliemia does not contraindicate their use in critically ill children [52]. Indeed, balanced crystalloids do not generate higher kalemia, because their [K^+^] content is rapidly diluted in the extracellular fluid and the [K^+^] shifting out of cells induced by normal saline-related hyperchloremic metabolic acidosis is much greater [53, 54]. Balanced crystalloids must be infused on a different route from red blood cell transfusions or Ceftriaxone as they contain calcium. Regarding side effects, balanced crystalloids should not be considered equivalent one to another and have their own adverse effects [55]. For example, lactate buffered balanced crystalloids may induce small rises in serum lactate concentration in patient with severe liver failure [56]. Similarly, high acetate concentrations have been associated with hypotension and direct myocardial toxicity [57]. Finally, balanced crystalloids do not generate additional costs in comparison with normal saline [58].

### Comparison between crystalloids and colloids

Although fluid bolus therapy with albumin seems to be non-inferior to crystalloids, its high cost, lack of availability, blood-borne infectious risk and high-chloride content do not allow its routine use to be recommended for initial fluid bolus therapy [13, 17], except in specific situations, such as severe dengue because of extensive capillary leaks [59]. Finally, other colloids should be abandoned in pediatric sepsis and other childhood circulatory failures [13, 17]. Starches use have been completely ruled out by the European Medicines Agency because of the risk of anaphylaxis, coagulopathy and acute kidney injury in adults [60] and gelatin have shown no benefit in the only RCT carried out in pediatric sepsis [61].

In summary, balanced crystalloids, which are closer to the composition of plasma with a lower chlorine content than normal saline, their theoretical reduction in the risk of hyperchloremic metabolic acidosis and subsequent acute kidney injury, inflammation, and coagulopathy are now suggested as the first-line choice for fluid bolus therapy in pediatric sepsis and other childhood circulatory failures.

## Fluid volume bolus and duration

Fluid bolus therapy is still recommended by the Surviving Sepsis Campaign in case of abnormal perfusion or hypotension for age in healthcare systems with access to intensive care [13], although related to excess mortality in a recent meta-analysis of 19 studies involving 9,321 cases of pediatric sepsis. This is likely due to that Yue et al. included mainly studies from low- to middle-income countries, such as India (n = 6), Kenya (n = 5) or Vietnam (n = 3). Moreover, this result was not consistent in the subgroup of general shock patients after exclusion of malaria case [62].

### Volume

The Surviving Sepsis Campaign recommends fluid boluses of 10–20 ml/kg of ideal body weight, up to 40–60 ml/kg over the first hour of management [13]. The European Resuscitation Council have recently extended this recommendation to all childhood circulatory failures, preferably with boluses of 10 ml/kg [17]. These smaller boluses comparing with previous guidelines [63, 64], are argued by the need for more frequent and faster clinical reassessment as well as the early use of vasoactive agents. However, it does not limit the total amount of fluid bolus therapy to be given. Even smaller volumes of 5–10 ml/kg may be beneficial to optimize preload in given pathologies such as pre-existing heart disease at risk of acute pulmonary edema, under the condition of even more frequent clinical reassessments [65]. This reconsideration of the 20 ml/kg paradigm is supported by a multicenter pilot study on 75 children with sepsis assigned to bolus of 10 ml/kg or 20 ml/kg after an initial bolus of 20 ml/kg for both groups, where no differences were found in mortality, hospital length of stay, PICU transfer, receipt of mechanical ventilation or inotropes and duration of mechanical ventilation [66]. The target of 40–60 ml/kg over the first hour of management through repeated boluses was associated with increased survival [25] and decreased occurrence of hepatomegaly [67] in pediatric historical studies. Finally, in adults, there are no prospective intervention comparing different volumes for initial resuscitation in sepsis [68] and one retrospective study showed that failure to receive 30 mL/kg of fluid bolus therapy within 3 h of sepsis onset was associated with increased mortality, delayed hypotension and ICU stay [69].

### Duration

The Surviving Sepsis Campaign recommends fluid boluses of 10–20 ml/kg of ideal body weight up to 40–60 ml/kg over the first hour of management in pediatric sepsis and no longer mentions the “5–10 min” duration as previously for fluid bolus therapy. We argue that this short duration should be maintained to achieve the above-mentioned goal of 1-h 40–60 ml/kg fluid bolus therapy even if no supportive studies in children clearly confirm the benefit in using 5 to 10 min rather than 10 to 20 min for fluid bolus therapy. A recent large multicentric Brazilian RCT with more than 10,000 critically ill adults found no difference in mortality after bolus at a rate of 999 ml/h versus 333 ml/h [70].

## Conclusion

Pediatric sepsis remains a significant global health threat, although revision to the reference 2005 children definition is still lacking. Management of pediatric sepsis have been updated by the 2020 Surviving Sepsis Campaign, whose compliance lead in decreasing sepsis-related mortality. These international guidelines notably suggest balanced crystalloids as the first-line solution for initial fluid bolus therapy. However, there is a very low level of evidence supporting this recommendation to date. Thus, the ideal fluid debate is not over, and the results of an ongoing large pediatric randomized clinical trial are eagerly awaited.

## Data Availability

Not applicable.
